# Thermally Engineered CVD for Controlling Crystal Orientation and Strain in Large-Area PtTe_2_ Layers

**DOI:** 10.3390/nano16120734

**Published:** 2026-06-13

**Authors:** Matteo Gardella, Alessandro Cataldo, Alessandro Forzinetti, Koushik Pasagadugula, Carlo S. Casari, Chiara Massetti, Christian Martella, Alessandro Molle, Alessio Lamperti

**Affiliations:** 1 CNR-IMM Unit of Agrate Brianza, Via C. Olivetti 2, I-20864 Agrate Brianza, Italy; matteo.gardella@mdm.imm.cnr.it (M.G.); alessandro.cataldo@mdm.imm.cnr.it (A.C.); alessandro.forzinetti@mdm.imm.cnr.it (A.F.); koushik.pasagadugula@mdm.imm.cnr.it (K.P.); chiara.massetti@mdm.imm.cnr.it (C.M.); alessandro.molle@cnr.it (A.M.); 2 Dipartimento di Chimica, Materiali e Ingegneria Chimica, Politecnico di Milano, P.zza Leonardo da Vinci 32, Edificio 6, I-20133 Milan, Italy; 3 Dipartimento di Energia, Politecnico di Milano, Via Lambruschini, I-20156 Milan, Italy; carlo.casari@polimi.it

**Keywords:** transition metal dichalcogenides, PtTe_2_, tellurization, grain orientation, wrinkling, strain, X-ray diffraction, Raman spectroscopy, chemical vapor deposition

## Abstract

Platinum ditelluride (PtTe_2_) is an emerging topological semimetal with intriguing optoelectronic properties. Scalable and controllable growth techniques are fundamental for its technological exploitation. Here, we synthesize large-area PtTe_2_ films by tellurization of pre-deposited platinum layers. By selectively modifying the tellurization parameters, we demonstrate the possibility of controlling the layer orientation of tellurized films and of introducing microscopic corrugation in the PtTe_2_ film. The first result is obtained by increasing the thermal budget of the process, which changes PtTe_2_ preferential crystalline orientation from (001) to (1−13)/(103) growth directions. The latter result is achieved by modifying the heating rate of the process at a fixed growth temperature equal to 550 °C. From the Raman analysis of a wrinkled sample, we find the coexistence of tensile and compressive strains depending on the corrugation site. The demonstrated control over grain orientation and microscopic corrugation provides a powerful strategy to tailor the structural and strain landscape of topological semimetals, providing a robust platform for strain engineering.

## 1. Introduction

Since the discovery of graphene, two-dimensional (2D) layered materials have revolutionized research in electronics, optoelectronics, and energy applications due to their unique physical and chemical properties [1,2]. Among these, Transition Metal Dichalcogenides (TMDs), characterized by the general formula MX_2_ (where M is a transition metal and X is a chalcogen), have emerged as a prominent class of materials. TMDs offer a versatile platform for engineering functional devices because their electronic properties—ranging from insulating to superconducting—can be precisely tuned by controlling the number of layers and the atomic arrangement within the lattice [3,4].

Platinum-based dichalcogenides, such as platinum ditelluride (PtTe_2_), have recently attracted significant interest. In its trigonal (1T) phase, PtTe_2_ is identified as a type-II Dirac semimetal, a topological state of matter featuring tilted Dirac cones in its electronic band structure [5]. This topological nature leads to intriguing electronic and optical properties, including anisotropic transport and intrinsic robustness protected by nontrivial topological invariants [6].

Realizing the technological potential of PtTe_2_ requires scalable growth methods capable of producing large-area films with controlled morphology [7,8]. Conventional chemical vapor deposition (CVD) often struggles with layer uniformity and precise lattice orientation control. A promising alternative for TMDs is a two-step process involving the sputtering of a solid metal precursor film followed by thermal tellurization [9,10]. Recent studies have demonstrated that by modulating the growth kinetics—such as temperature ramps, precursor thickness, and gas flow rates—it is possible to tailor the orientation of 2D layers from horizontal to vertical alignments [7,8]. For example, the transition from horizontal layer-by-layer growth to vertical orientation can be driven by the accumulation of internal strain during the expansion of the PtTe_2_ lattice [7].

The ability to control the surface structure and grain orientation is of paramount interest for catalysis. Edge sites and defects of TMDs are known to be significantly more active than their basal planes for various reactions [11,12]. In PtTe_2_, orientation engineering has been shown to synergistically boost the oxygen reduction reaction; vertically aligned layers expose dangling bond-rich edges that promote O_2_ adsorption, while horizontal layers facilitate O−O bond breaking [13,14]. Furthermore, the introduction of surface defects, such as tellurium vacancies, and the engineering of grain boundaries provide additional active sites for both electrocatalytic reactions (like the hydrogen evolution reaction) and photocatalytic applications, such as water splitting [15].

Finally, the mechanical response of TMDs to external and internal stresses introduces the phenomenon of corrugation or wrinkling [16]. High tensile strain can be incorporated during the synthesis due to the mismatch of thermal expansion coefficients between the 2D film and the growth substrate. In the case of PtTe_2_ grown on dielectric substrates like silica, fast thermal cycles can induce significant thermal induced strain, which is released through the formation of macroscopic wrinkles [17]. While such corrugations can be detrimental to device uniformity, they also offer a novel opportunity for material engineering, enabling the development of “wrinkled electronics” and sensors that leverage localized changes in the band structure and surface area [18,19]. Unlike trivial materials where the strain is primarily utilized to modulate the bandgap or carrier mobility, the strain engineering in topological materials like PtTe_2_ enables the manipulation of symmetry-protected Dirac states and the driving of quantum phase transitions [20,21]. Localized deformations can thus act as functional platforms for the reversible control of anisotropic transport and the deterministic “on/off switching” of topological states, offering a pathway toward high-speed, low-dissipation quantum electronics [22]. Understanding and optimizing the tellurization process is therefore crucial for achieving either stress-free growth or controlled corrugation in high-quality PtTe_2_ films.

In this work, we use a two-step process for growing large-area PtTe_2_ films on standard silicon substrates. By increasing the thermal budget of the tellurization process, we study its influence on the resulting PtTe_2_ orientation, showing a clear change from in-plane to out-of-plane preferential orientation, as demonstrated by the XRD patterns showing a transition from strong (001) to complete (1−13)/(103) preferential growth direction. Alternatively, by varying the kinetics of the process, we propose a route to induce progressive wrinkling of PtTe_2_ films, and we use Raman mapping to investigate the influence of such corrugations on the material properties. These findings demonstrate the possibility of tailoring the properties of the PtTe_2_ layers using a scalable growth process, paving the way for an easily tunable growth depending on the required application field.

## 2. Materials and Methods

###  2.1. Text Generation 

Part of the text included in the Section 1 has been generated using Google NotebookLM (powered by Gemini 3.1 model), starting with a selected set of references and a given outline to drive the text. The authors have reviewed and edited the output and take full responsibility for the content of this publication.

### 2.2. Synthesis of PtTe_2_ Films

Large-area platinum precursor films are deposited on standard silicon substrates by DC magnetron sputtering (Kurt J. Lesker Company, Jefferson Hills, PA, USA) of a platinum target, using Ar as process gas and a power of 100 W.

Tellurium lumps (~160 mg) and platinum films are placed in the center of the upstream furnace of a 2 inch dual-zone tubular furnace (PlanarTECH Ltd., Cambridge, UK) and processed at a high temperature (450–550 °C) and low pressure (20 torr). A 100 sccm Ar + H_2_ 4% mixture is used as carrier gas during the whole process. Different thermal ramps are used for each sample, as described in Section 3.

### 2.3. Characterization of PtTe_2_ Films

Raman spectra and Raman maps were acquired using a InVia analyzer (Renishaw Ltd., Wotton-under-Edge, UK) coupled with a confocal microscope equipped with a 514 nm laser.

XPS measurements were conducted with an ESCA 5600 system (Physical Electronics Inc., Chanhassen, MN, USA), using a monochromatic Al Kα x-ray source (1486.6 eV) and a concentric hemispherical analyzer. The spectra were recorded at a 45° take-off angle and were referenced to the C-1s peak at 285 eV.

XRD patterns were acquired in a θ–2θ configuration using an HRXRD IS2000 four-circle goniometer (Italstructure, Riva del Garda, Italy) equipped with a Cu Kα radiation (λ = 1.5406 Å) and a curved 120° position-sensitive detector (CPS-120, Inel, Artenay, France).

SEM images were acquired in a secondary electron signal using a Supra 40 SEM (Zeiss, Oberkochen, Germany) with an electron high tension of 15 kV.

AFM images were acquired using a NX10 system (Park Systems, Gwacheon City, South Korea) equipped with ultra-sharp silicon probes (nominal tip radius < 10 nm) in tapping mode.

### 2.4. Python 3.12.11 Scripts for Data Analysis

The morphological and spectroscopic data (AFM analysis and Raman maps) were processed using custom-made Python scripts developed by the first author. The algorithms, designed to compute local curvature and statistical distributions, were implemented within the Spyder 6.1.4 IDE. Large Language Model (LLM) interaction via Google Gemini 1.5 Pro was utilized as a collaborative tool for code optimization and the refinement of mathematical models for surface analysis. Scripts are archived on Zenodo (https://doi.org/10.5281/zenodo.20137357).

## 3. Results and Discussion

### 3.1. Thermal Budget Influence on PtTe_2_ Crystalline Orientation

Large-area platinum ditelluride (PtTe_2_) films were grown through a two-step process already described in a previous work and briefly recalled in the Section 2 [17]. In this study, we will consider the PtTe_2_ films obtained by tellurization of 8 nm thick Pt layers deposited on standard Si/SiO_2_ substrates with sizes of about 2 × 1 cm^2^. Each sample was then tellurized with a different thermal process in a tellurium-enriched atmosphere using a tubular furnace.

**Figure 1:**
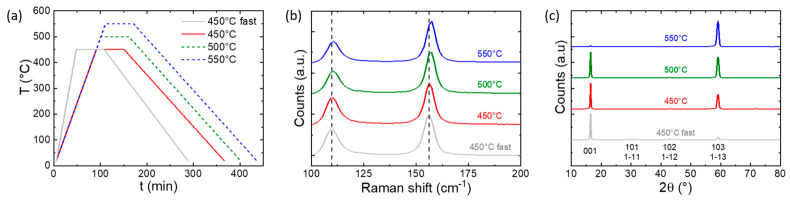
(**a**) Tellurization thermal ramps used for the first set of samples by increasing the thermal budget; (**b**) Raman spectra and (**c**) XRD patterns acquired for the samples tellurized using the different processes.

In the first set consisting of four samples, we modified the tellurization process to study the effect of the increase in the thermal budget on the morphology of the PtTe_2_ films. The thermal ramps are reported in Figure 1a. The first sample was tellurized using our original process at 450 °C with a heating rate of 10 °C/min and an initial cooling rate of 2.4 °C/min (gray curve). For the second sample, we increased the thermal budget by reducing the heating rate to 5 °C/min and the cooling rate to 2 °C/min (red curve). This reduction was functional in view of increasing thermal budget by growth temperature increase, as the wrinkling of the PtTe_2_ films was observed in the case of high thermal stress in the tellurization process [17]. The third sample was then tellurized at 500 °C (green curve) and the fourth one was tellurized at 550 °C (blue curve).

Samples grown utilizing different processes were initially characterized by Raman spectroscopy, as reported in Figure 1b. In all cases, two peaks can be clearly identified at about 110 cm^−1^ and 156 cm^−1^, respectively, corresponding to the in-plane (E_g_) and out-of-plane (A_1g_) vibrational modes characteristic of the 1T phase of the PtTe_2_. Peak position values of different samples are reported in Table 1. Apart from a slight blue shift of the modes as the thermal budget increased, all spectra point to a successful tellurization of the Pt precursor film. The peak position values fall well within the variation range expected from literature data for bulk PtTe_2_ samples obtained by different techniques, as reported in the table [23,24,25,26].

To study the chemical composition of the resulting PtTe_2_ layers, X-Ray photoemission spectroscopy (XPS) characterization was performed on the lowest thermal budget sample. The acquired spectrum is reported in Supplementary Materials Section S1. In agreement with similar films in literature, the sample features pronounced superficial oxidation on the Te-3d core levels, while Pt-4f signal only generates from Pt-Te bonds [27,28]. The calculated stoichiometry of the PtTe_2.59_ points toward the complete tellurization of the film, with an additional condensation on the surface of tellurium excess. As PtTe_2_ degradation and Te loss is only expected at temperatures above 600 °C, similar stoichiometry is expected for the other samples [27,29].

The structural properties of the PtTe_2_ films were then investigated by means of X-ray diffraction (XRD). The collected XRD patterns are reported in Figure 1c. The diffraction pattern for the sample tellurized using the original process (gray curve) is dominated by the (001) peak located at 2θ = 17°, pointing to an in-plane preferential orientation, where the crystalline cell basal plane lies parallel to the substrate surface. Although much less intense, additional peaks are visible at 2θ = 31°, 2θ = 43°and 2θ = 58°, respectively, corresponding to the (1−11)/(101), (1−12)/(102) and (1−13)/(103) peaks. For the samples tellurized using the slower processes at 450 °C and 500 °C (red and green curves), the diffraction patterns show a progressive increase in the (1−13)/(103) peak, which becomes comparable to the (001) peak. The trend is confirmed by the tellurization process run at 550 °C (blue curve), for which the (1−13)/(103) peak becomes the dominant diffraction peak, while the peaks related to other crystalline directions are suppressed. These results suggest a strong correlation between the thermal budget of the tellurization process and the crystalline orientation of PtTe_2_ layers. Remarkably, whereas a grain orientation depending on the initial Pt thickness was reported elsewhere, here we demonstrate that optimization of the thermal process can as well allow for orientation engineering without being limited by the initial metal precursor [13].

**Figure 2:**
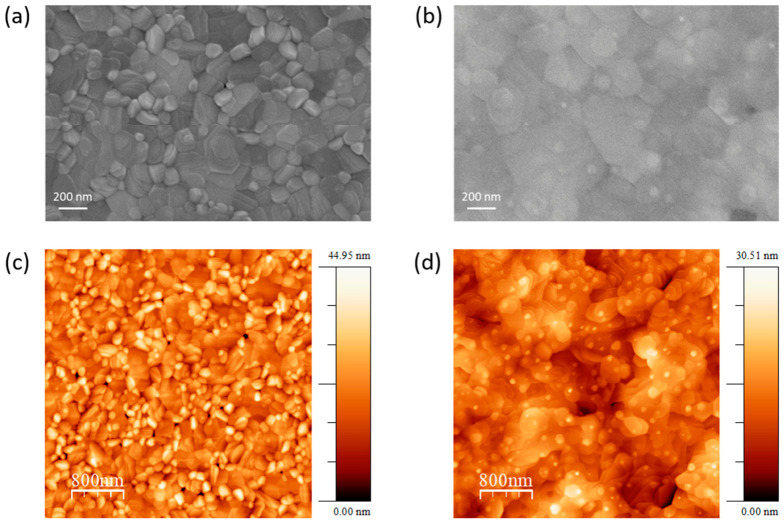
(**a**,**b**) SEM and (**c**,**d**) AFM images acquired from the samples tellurized with the lowest and the highest thermal budget processes, respectively.

To evaluate the influence of the thermal budget on the PtTe_2_ films surface morphology, we performed a Scanning Electron Microscopy (SEM) focusing on the samples tellurized with the lowest and the highest thermal budget (gray and blue curves in Figure 1). The SEM image of the sample tellurized with the fast process at 450 °C, reported in Figure 2a, reveals a heterogeneous distribution of highly geometrical grains qualitatively differing in size and orientation, where the smaller grains seem to be those with a pronounced out-of-plane tilt. Conversely, the SEM image of the sample processed at 550 °C, reported in Figure 2b, shows a different morphology. The number of small grains is drastically reduced compared to the previous case, while the larger grains have a more uniform orientation that results in a reduced image contrast.

SEM images for the samples tellurized with intermediate thermal budgets are reported in Supplementary Materials Section S2. Considering the full set of samples, a coherent surface morphology evolution can be observed as the thermal budget is increased. In fact, smaller grains merge in larger structures where grain boundaries become more difficult to identify and more regular atomic terraces are formed.

We then used the Atomic Force Microscopy (AFM) to gather more quantitative information on the PtTe_2_ surface morphology for the two extremal samples. The AFM image acquired on the sample tellurized with the fast process at 450 °C, shown in Figure 2c, visually confirms the conclusions inferred from the SEM characterization. From this image, a roughness of 5.6 nm is extracted, while a total thickness of 42 nm is established from a scratch in the film (see Supplementary Materials Section S3). Even for the sample tellurized at 550 °C, the AFM topography reported in Figure 2d qualitatively agrees with the SEM characterization, featuring more uniform grains with evident formations of atomic terraces, whose topmost and narrower layers account for the bright features observed at the apex of the grains. Remarkably, for this sample the roughness was reduced to 3.8 nm despite a total thickness increase to 60 nm (see Supplementary Materials Section S3). Thickness variation between the two samples is not expected to arise from different tellurium incorporation, as the tellurization is conducted in a temperature range where full tellurization was demonstrated by XPS and the degradation mechanisms were not thermally activated yet [27,29]. Rather, it could have depended on the different preferential growth direction of the grains, as assessed by the XRD characterization.

We made a more detailed comparison between the two morphologies by estimating the average grain size. Due to the low-contrast fine texture of the films, we used the intercept method. For each AFM image, we traced 10 horizontal lines and 10 vertical lines, along which we counted the number of intersected grains (*P*). The average grain size (*d*) can then be defined as:

$$d=\frac{n\cdot L}{P}$$

where *n* is the number of lines (20) and *L* is the length of the lines (4 µm). The AFM images with overlapped grid lines (and calculated number of intersected grains for each line) and average grain size calculations are reported in Supplementary Materials Section S4. The resulting values show that the estimated average size increases from 150 nm to 300 nm by increasing the thermal budget of the tellurization process.

### 3.2. Heating Rate Influence on PtTe_2_ Wrinkling

**Figure 3:**
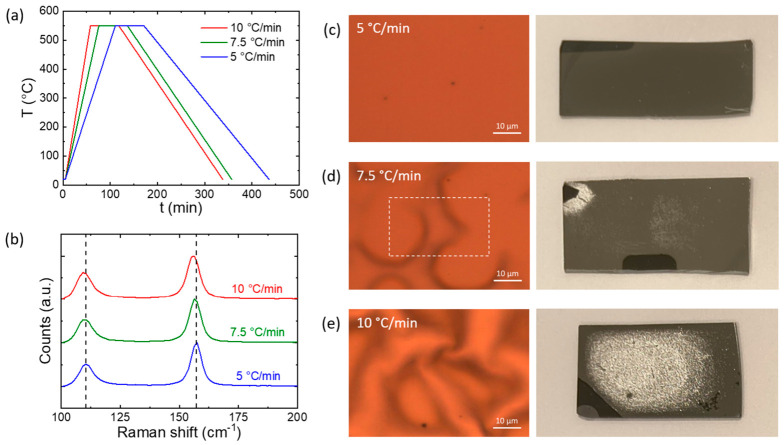
(**a**) Tellurization thermal ramps used for the second set of samples by changing the heating rate; (**b**) Raman spectra for the samples tellurized at different heating rates; (**c**–**e**) optical images of samples tellurized at increasing heating rates (from top to bottom); for each process, a photograph of the sample showing increasing wrinkling is reported on the right side.

In the second set of experiments, we kept the growth step temperature constant at 550 °C and we increased the heating rate of the process to study the kinetic effects on the tellurization of three different samples. The thermal ramps are reported in Figure 3a: starting from the process already presented in Section 3.1 with a heating rate of 5 °C/min (blue curve), this value was increased to 7.5 °C/min (green curve) and 10 °C/min (red curve).

As shown in Figure 3b, the Raman spectra acquired for the three samples all show the PtTe_2_ characteristic vibrational modes, demonstrating successful tellurization of the Pt precursor films despite the increasing heating rate, which only results in a progressive red shift of the peaks. Table 2 reports the modes positions of the three samples: the E-mode shifts from 110.7 cm^−1^ to 109.9 cm^−1^ as the rate is increased, while A-mode simultaneously shifts from 157.1 cm^−1^ to 155.9 cm^−1^.

However, upon visual inspection of the samples, the increase of the heating rate also induced a macroscopic variation in the different PtTe_2_ films, as evident from the photographs shown on the right side of Figure 3c–e. The sample tellurized at 5 °C/min (Figure 3c) presents a homogeneous surface coverage that looks reflective and flat. When the heating rate is increased to 7.5 °C/min (Figure 3d), macroscopic defects appear in the central part of the sample, and become visible as white spots. A particularly dense concentration of these defects is observed in the top right corner of the sample. Finally, when the rate is further increased to 10 °C/min (Figure 3e), the whole PtTe_2_ film is affected by similar defects.

To investigate the nature of these defects, we acquired optical microscopy images of the different samples, as reported on the left side of Figure 3c–e. We thus found that the defects are extended microscopic wrinkles that can be ascribed to the thermal stress accumulated during the tellurization process when the heating rate is increased. Indeed, a homogeneous surface is observed for the 5 °C/min process, whereas optical microscope images reveal a significant densification of wrinkles of the samples tellurized using 7.5 °C/min and 10 °C/min heating rates. Based on these results, the critical heating rate threshold for wrinkle onset on Si/SiO_2_ substrates in the tellurization process at 550 °C is bound within 5 °C/min and 7.5 °C/min. However, this threshold is not only related to the heating rate, but also to the substrate and the tellurization temperature. For instance, a similar wrinkling formation was observed on fused silica substrates for heating rates well above 10 °C/min in the tellurization processes at 450 °C, thus demonstrating the possibility to generalize this kinetic wrinkling route [17].

In the following, we will focus on the wrinkle highlighted by the dashed box in Figure 3d, related to the sample tellurized at the 7.5 °C/min heating rate. This wrinkle is of particular interest as it is surrounded by flat regions of PtTe_2_, offering the possibility to easily compare the properties of corrugated and flat material.

**Figure 4:**
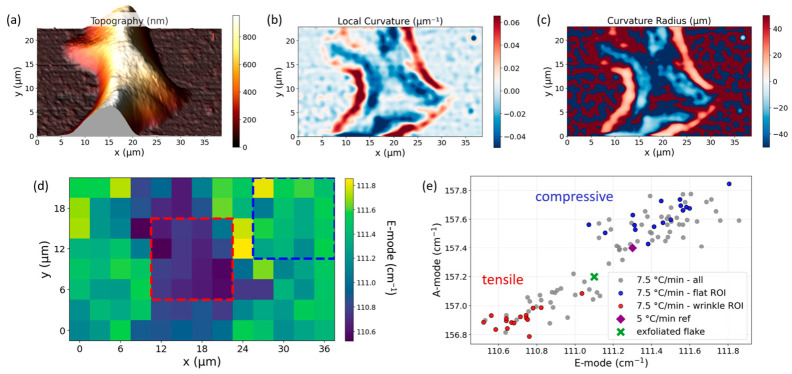
(**a**) 3D visualization of the wrinkle AFM topography image; (**b**,**c**) local curvature and curvature radius maps extracted from AFM image; (**d**) Raman map of the E mode acquired in correspondence with the wrinkle; dashed squares highlight the regions of interest on the wrinkle (red) and on a flat region (blue); (**e**) overall dispersion of the peak positions in the Raman map spectra (gray dots), showing clear distinction between flat (blue dots) and wrinkle regions due to different strains; a spectrum from an exfoliated bulk flake is reported as a reference for unstrained material (green cross).

The morphology of the selected wrinkle was investigated by means of AFM. A 3D visualization of a topography image of roughly 40 × 25 µm^2^ is reported in Figure 4a. The wrinkle has a vertical dynamic of approximately 900 nm over a length of about 15 μm. Local curvature and curvature radius maps calculated from this image are shown in Figure 4b,c. An arbitrary cut-off of the curvature radius was set at 50 μm to better highlight the curved regions with respect to the flat ones. The wrinkle presents a relatively flat crest which connects the two sides, featuring a curvature radius on the order of about 20 μm, which is reflected in curvature values on the order of 0.05 μm^−1^.

To study the effects of corrugation on the material properties, we acquired a Raman map across the wrinkle with size 36 × 21 µm^2^ (3 µm steps, 13 × 8 point spectra), focusing on the spectral shift of the PtTe_2_ vibrational modes. In Figure 4d, the Raman shift map of the E mode is shown. Remarkably, the map shows a strict correlation with the wrinkle morphology, showing a red-shifted E mode in correspondence with the wrinkle with respect to the surrounding flat regions. A similar conclusion is conveyed by the A-mode map reported in Supplementary Materials Section S5.

The Raman map was then analyzed in deeper details by calculating the relative position of the E and A modes to create the dispersion plot reported in Figure 4e, where each point of the map is represented by a gray dot with coordinates given by the E and A modes Raman shifts. The resulting graph strongly suggests the presence of two separate distributions, which can be roughly distinguished for A mode position below or above 157.3 cm^−1^.

To establish a clear connection between the spectra dispersion and their spatial positioning within the map, we focused on two specific regions of interest (ROIs), as highlighted in Figure 4d: the red dashed square shows a 4 × 4 subset of spectra that have been acquired on the wrinkle, while the blue dashed square shows a 4 × 4 subset of spectra that have been acquired on a flat PtTe_2_ region. The points originating from these two subsets have been overlaid in Figure 4e by red and blue dots, respectively, clearly demonstrating that the red-shifted dispersion is ascribed to spectra acquired on the wrinkle, whereas the blue-shifted dispersion originates from the spectra acquired on the flat part.

To understand the meaning of the obtained distribution, we included two additional pieces of data in the dispersion plot. The first one (green cross) indicates the position of a strain-free PtTe_2_, which we assume to be the case for a flake that was mechanically exfoliated from a bulk single crystal synthesized via the melt growth method [24]. Remarkably, the added reference almost perfectly represents a cross-over point between the two separate dispersions, pointing towards the presence of opposite strains in the flat and in the wrinkle ROIs. The second point added to the plot (purple diamond—see Supplementary Materials Section S6) is instead related to the sample tellurized with the slower heating rate of 5 °C/min, which did not form wrinkles (Figure 3c). The data is collocated in a position corresponding to a slightly compressive strain, which is reasonable considering the expansion factor from Pt precursor to tellurized PtTe_2_ layers. Therefore, we must distinguish two different contributions in the accumulated strain: the first is intrinsic due to lattice expansion, while the second can be engineered by changing the kinetics of the process. A different intrinsic contribution could be expected for epitaxial growth techniques due to lattice mismatch with the substrate, but this is not the case as the films are grown on the amorphous silica capping layer of silicon wafer. If we now consider the compressive strain observed in the flat ROI for the wrinkled sample, we can notice that the average blue-shift is higher. This result is consistent with the ultimate wrinkling of the film to release the excessive compressive strain.

In contrast with other 2D materials, for which the influence of strain on Raman modes is extensively studied, we are not aware of similar considerations for PtTe_2_ [30,31]. In analogy with other materials, we can attribute the red-shift observed on the wrinkle to a tensile strain, which indeed is expected to be most pronounced on the crest [20,32,33]. By applying a conventional strain model for 2D materials, the maximum tensile strain ε (assumed to be uniaxial) on top of the crest can be estimated using the following equation [20]:

$$\varepsilon =\frac{\pi^{2}hd}{\left(1-\nu^{2}\right)l^{2}}$$

where h and l are the wrinkle height and length, d is the film thickness and ν is the material Poisson’s ratio. Analyzing a representative wrinkle line profile shown in Supplementary Materials Section S7, we have h = 810 nm and l = 15.1 µm. Assuming from equivalent tellurizations d = 60 nm, and we assume ν = 0.26 based on ref [34]. As a result, we obtain a maximum strain ε ~ 0.23%, which is a reasonable value compared to similar works on 2D materials [20,35].

In conjunction with the tensile strain observed on the wrinkle, the comparison with the unstrained exfoliated flake shows that the flat region in our sample is not relaxed but rather shows a compressive strain, with a blue-shift comparable in magnitude to the red-shift observed on the corrugation. Although counterintuitive, this evidence could be explained just by considering that the out-of-plane wrinkling could indeed be a way to release the excess compressive strain accumulated during the tellurization process due to the lattice expansion.

## 4. Conclusions

In summary, we conducted a parametric variation of the tellurization process of platinum ultrathin films to investigate the thermal influence on the resulting PtTe_2_ properties, focusing on crystallographic orientation and microscopic morphology.

First, by increasing the tellurization temperature, we demonstrated a route to control grain orientations as assessed by XRD characterization. This result is potentially relevant for electro- and photocatalysis applications, where a different reactivity is expected depending on the exposed surface.

Second, by increasing the heating rate of the tellurization process, we exploited the difference in thermal conductivity between PtTe_2_ and the substrate to induce in a controlled way the formation of microscopic wrinkling. By Raman mapping, we prove a strong correlation between morphology and vibrational modes dispersion, paving the way to strain engineering studies on the material.

These results demonstrate the potential of our two-step growth process, offering the possibility to tailor PtTe_2_ properties by a simple process modification. Development of a scalable and versatile synthesis technique is crucial for full technological exploitation of the material.

## Figures and Tables

**Table 1:** PtTe_2_ vibrational modes position for different thermal budgets and literature comparison.

Process	E-Mode (cm^−1^)	A-Mode (cm^−1^)	Reference
450 °C fast	110.0	156.1	this work
450 °C	110.2	156.4	this work
500 °C	110.7	157.0	this work
550 °C	110.7	157.1	this work
Tellurization	112.4	158.1	[23]
Exfoliation	111.1	157.2	[24]
Eutectic synthesis	110.7	157.2	[25]
CVD	109.0	154.7	[26]

**Table 2:** PtTe_2_ vibrational modes position for different heating rates.

Heating Rate	E-Mode (cm^−1^)	A-Mode (cm^−1^)
5 °C/min	110.7	157.1
7.5 °C/min	110.3	156.6
10 °C/min	109.9	155.9

## Data Availability

Dataset available from the authors upon reasonable request.

## Supplementary Materials

The following supporting information can be downloaded at: https://www.mdpi.com/article/10.3390/nano16120734/s1, Section S1: XPS characterization; Section S2: Additional SEM characterization; Section S3: Films thickness determination; Section S4: Grain size–intercept method; Section S5: A-mode Raman map; Section S6: Additional Raman spectrum; Section S7: Wrinkle AFM line profile.

## Abbreviations

The following abbreviations are used in this manuscript:

2D	Two-dimensional
AFM	Atomic force microscopy
CVD	Chemical vapor deposition
PtTe_2_	Platinum ditelluride
ROI	Region of interest
SEM	Scanning electron microscopy
TMD	Transition metal dichalcogenide
XPS	X-ray photoemission spectroscopy
XRD	X-ray diffraction
