# Supplementary material for: Thermally Engineered CVD for Controlling Crystal Orientation and Strain in Large-Area PtTe2 Layers
Source: Nanomaterials (Basel). 2026 Jun 13;16(12):734. doi: 10.3390/nano16120734 (PMC13305040; doi:10.3390/nano16120734)
Supplement: Supplementary file 1 [file nanomaterials-16-00734-s001.zip › nanomaterials-4347911-supplementary.pdf]

nanomaterials

MDPI

Supplementary Materials

Thermally Engineered CVD for Controlling Crystal Orientation and Strain in Large-Area $PtTe_2$ Layers

Matteo Gardella [1], Alessandro Cataldo [1,2], Alessandro Forzinetti [1,3], Koushik Pasagadugula [1,3], Carlo S. Casari [3], Chiara Massetti [1], Christian Martella [1,*], Alessandro Molle [1] and Alessio Lamperti [1,*]

[1] CNR-IMM Unit of Agrate Brianza, Via C. Olivetti 2, I-20864 Agrate Brianza, Italy; matteo.gardella@mdm.imm.cnr.it (M.G.); alessandro.cataldo@mdm.imm.cnr.it (A.C.); alessandro.forzinetti@mdm.imm.cnr.it (A.F.); koushik.pasagadugula@mdm.imm.cnr.it (K.P.); chiara.massetti@mdm.imm.cnr.it (C.M.); alessandro.molle@cnr.it (A.M.)

[2] Dipartimento di Chimica, Materiali e Ingegneria Chimica, Politecnico di Milano, P.zza Leonardo da Vinci 32, Edificio 6, I-20133 Milan, Italy

[3] Dipartimento di Energia, Politecnico di Milano, Via Lambruschini, I-20156 Milan, Italy; carlo.casari@polimi.it

* Correspondence: christian.martella@cnr.it (C.M.); alessio.lamperti@cnr.it (A.L.)

S1 – XPS characterization

The sample tellurized with the lowest thermal budget process was characterized by means of XPS. In **Figure S1**, the spectral regions corresponding to Te-3d, O-1s and Pt-4f core levels are reported. A strong oxidation is observed on the tellurium signal while on platinum they show a single component related to Pt-Te bonding. The calculated stoichiometry of PtTe2.59 is compatible with a full tellurization of the Pt precursor followed by a possible surface condensation of excess tellurium, which would also explain the high oxidation grade in our sample.

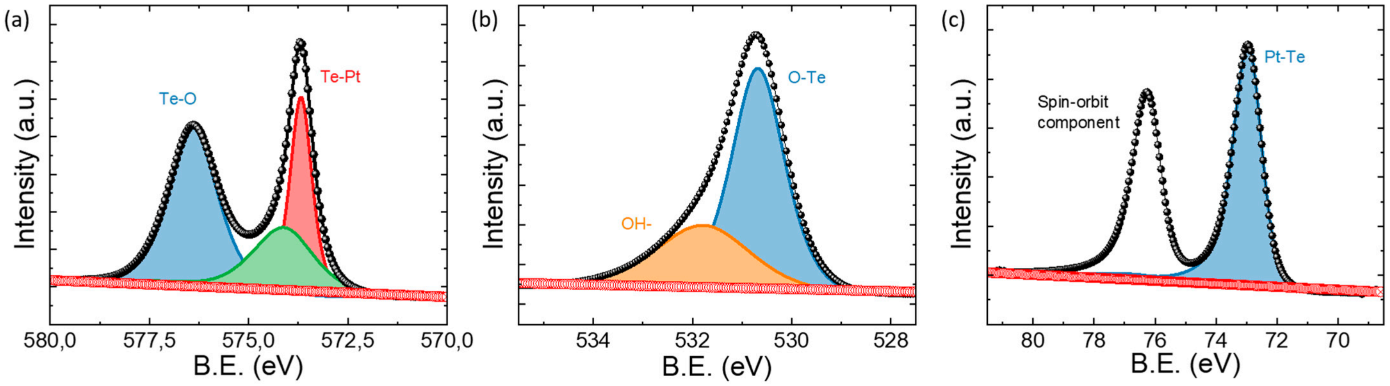

Figure S1. XPS characterization. a) Te-3d$_{5/2}$, b) O-1s and c) Pt-4f core levels.

S2 – Additional SEM characterization

SEM images of the samples tellurized with two intermediate thermal budget processes are shown in Figure S2. Together with the SEM images reported in Figure 2 complete the set of data, showing a morphological evolution as the thermal budget of the tellurization process is increased.

 https://doi.org/10.3390/nano16120734

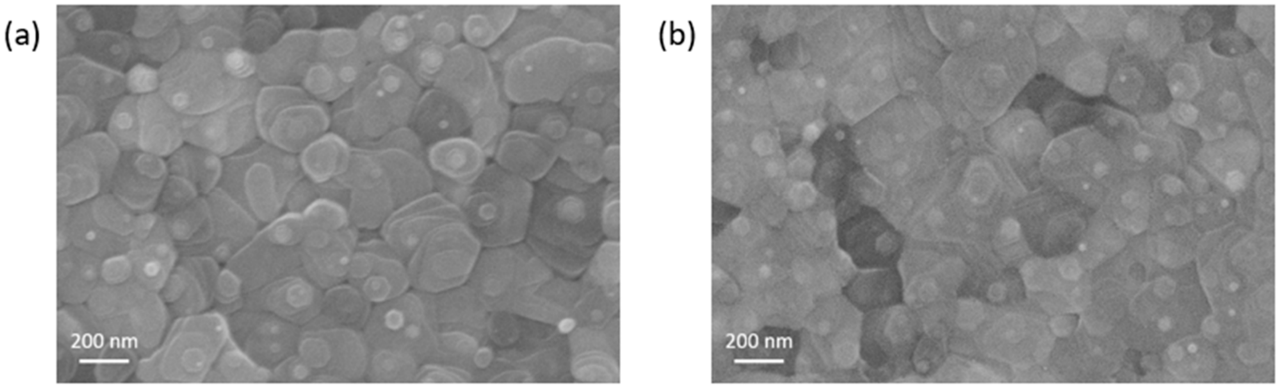

Figure S2. SEM images of the samples tellurized with intermediate thermal budgets at (a) 450 °C and (b) 500 °C.

S3 – Films thickness determination

$PtTe_2$ films were scratched to determine their thickness. Figure S1a (AFM image) and S1b (heights histogram) refer to the sample tellurized using the lowest thermal budget process, showing a thickness of about 42 nm established from distance between the centres of the two distribution peaks. Similarly, Figure S1c (AFM image) and S1d (heights histogram) refer to the sample tellurized using the highest thermal budget process, showing a thickness of about 60 nm.

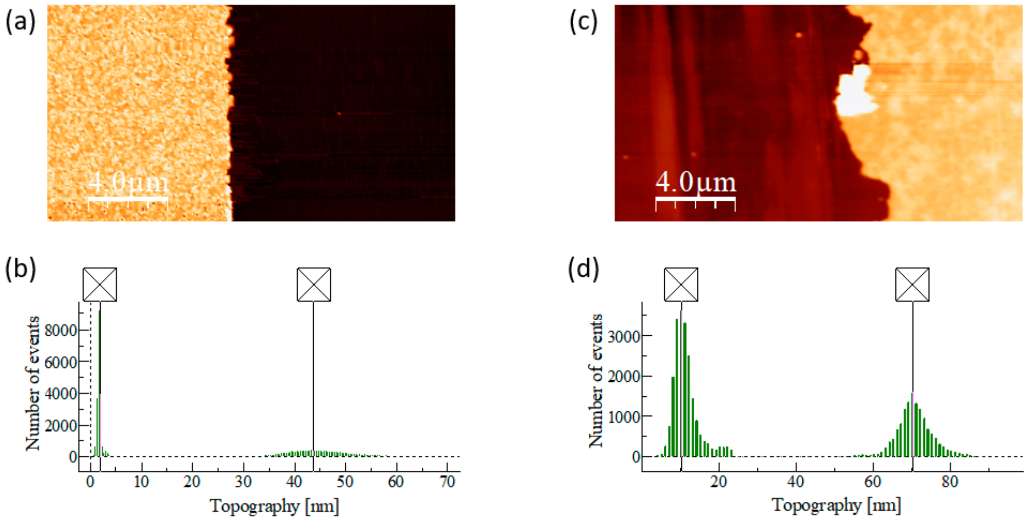

Figure S3. AFM images (a,c) and corresponding height histograms (b,d) for the samples tellurized using the lowest thermal budget process and the highest thermal budget process, respectively.

S4 – Grain size - intercept method

As shown in Figure S2, a grid of 10 × 10 lines was superimposed on the topography AFM images reported in the main text (Figure 2b and 2d). Along each line, the number of intercepted grains (p) was manually counted as reported by red numbers. The sum of all these values is the P parameter used in the calculation of average grain size (as described

in the main text). Due to possible misinterpretation of atomic terraces as grain boundaries, for each image we estimate a large relative error calculated as:

$$\varepsilon_r = 100 \times \frac{p_{max} - p_{min}}{p_{avg}}$$

where p_{max} and p_{min} are the maximum and minimum number of grains intercepted on a single line, and p_{avg} is the average number of grains intercepted on each line.

Under these approximations, we found d = 151 ± 57 nm for the lowest thermal budget tellurization process (Figure S2a, P = 529) and d = 305 ± 162 nm in case of the highest thermal budget process (Figure S2b, P = 262). Despite the large errors, these results clearly show a net grain size increase.

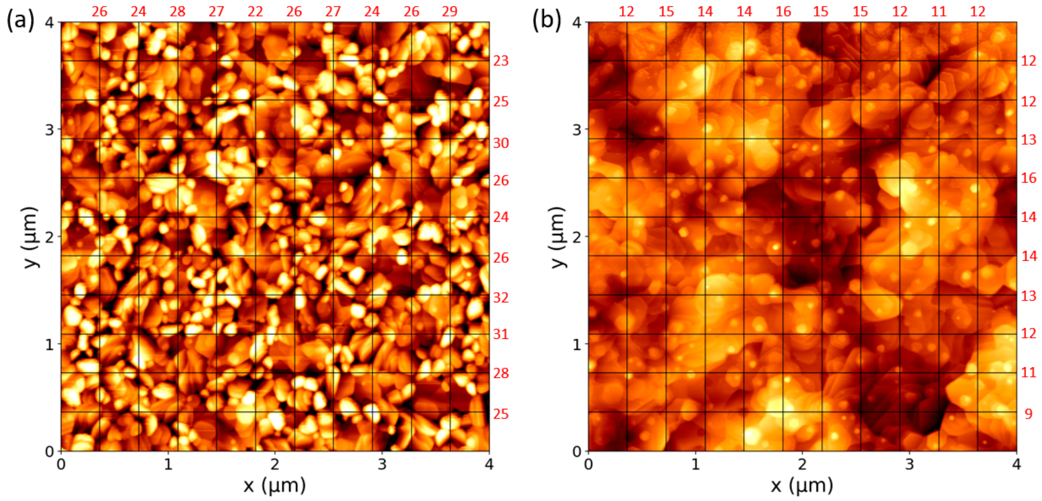

Figure S4. Grid lines superimposed on AFM images for the lowest thermal budget process (a) and highest thermal budget process (b).

S5 – A-mode Raman map

Together with the E-mode map shown in the main text (Figure 4d), A-mode in correspondence of the $PtTe_2$ wrinkle was mapped as well, as reported in Figure S3. The A-mode map is in qualitative agreement with E-mode map, clearly resembling the wrinkle shape.

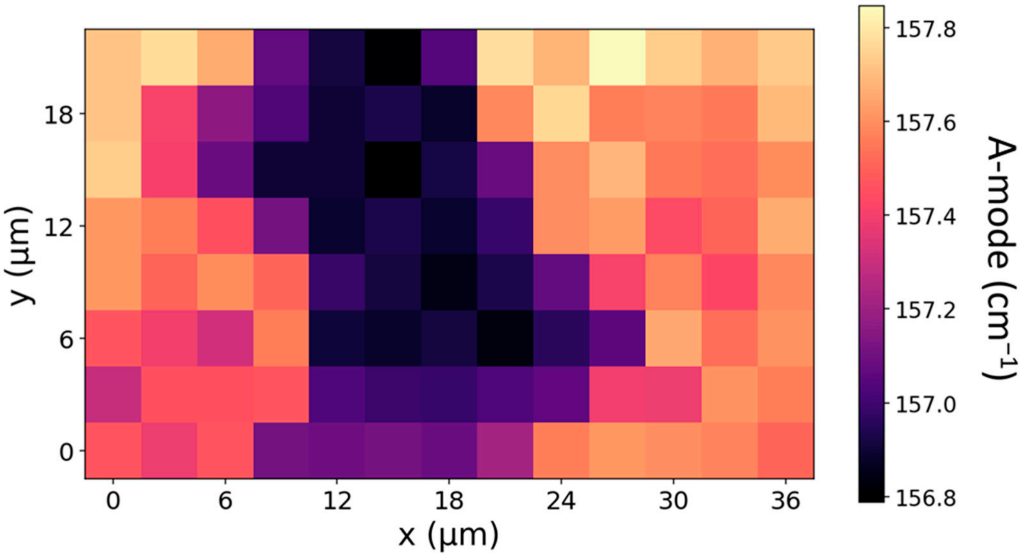

Figure S5. A mode Raman map in correspondence of the wrinkle.

S6 – Additional Raman spectrum

A new spectrum for the non-wrinkled sample tellurized with the 5 °C/min heating rate was acquired using the same acquisition parameters used for the Raman map of the wrinkle. The spectrum is reported in Figure S6: E-mode is located at 111.3 cm^{-1} and A-mode is located at 157.4 cm^{-1}. This data is added to the dispersion plot in Figure 4e as a reference for the intrinsic compressive strain accumulated during the tellurization process.

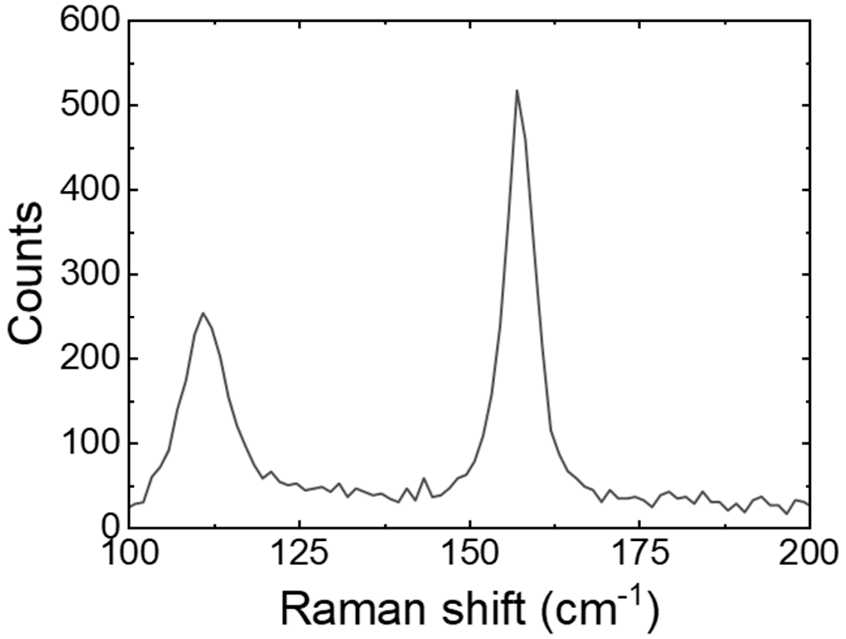

Figure S6. Additional Raman spectrum of the sample tellurized with the 5 °C/min heating rate.

S7 – Wrinkle AFM line profile

We selected a representative profile of the wrinkle AFM image as shown in Figure S5. From the extracted line profile, we determined the wrinkle height and length values used for the estimation of maximum uniaxial strain on top of the crest, as described in main text.

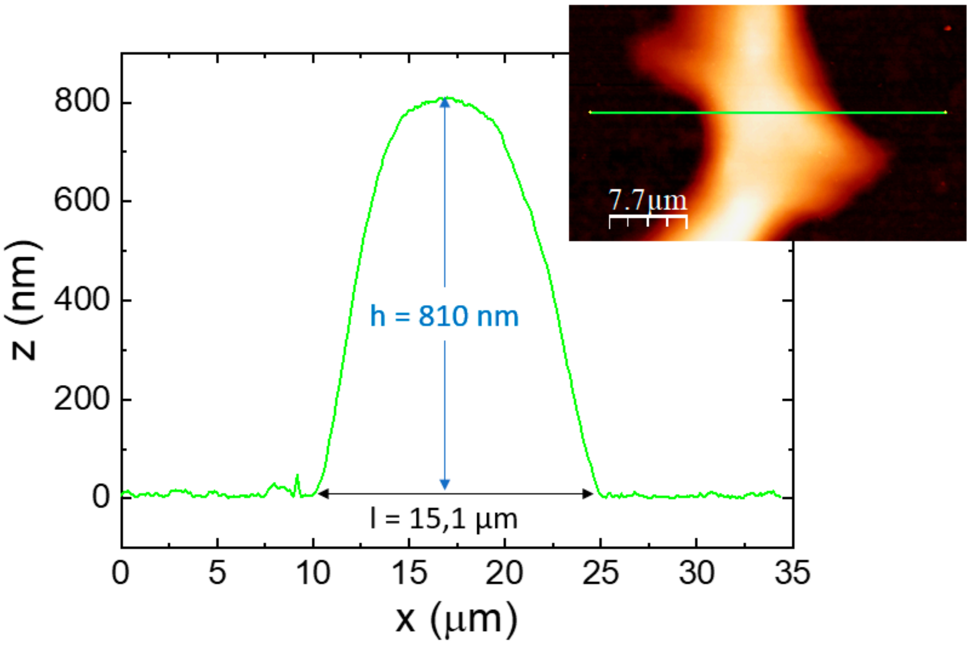

Figure S7. Representative wrinkle line profile. Inset shows the selected line on the 2D topography image of the wrinkle.

https://doi.org/10.3390/nano16120734
